# Adult-Onset Still's Disease With Normal Ferritin Levels and Severe Sulfasalazine-Induced Probable Case of Drug Reaction With Eosinophilia and Systemic Symptoms (DRESS) Syndrome: A Unique Presentation and Management Challenges

**DOI:** 10.7759/cureus.76723

**Published:** 2025-01-01

**Authors:** Lekhana Dayanand, Rahil A.Y., Rohan Krishna NK

**Affiliations:** 1 Internal Medicine, Fortis Hospital, Rajajinagar, Bengaluru, IND

**Keywords:** adult-onset still's disease, complications, dress syndrome, normal ferritin, nsaids, rash, steroids, sulfasalazine, urticarial vasculitis, yamaguchi criteria

## Abstract

Adult-onset Still's disease (AOSD) is an uncommon clinical condition with an uncertain cause, characterized by arthritis, fever, evanescent rash, and other systemic presentations. This case report describes a 26-year-old female who had a fever, arthralgia, vomiting, sore throat, bilateral distal extremities edema, hypertension, and normal ferritin. She was diagnosed with AOSD using the Yamaguchi criteria based on exclusion and was treated with hydroxychloroquine and sulfasalazine. The patient's arthralgia improved significantly. However, she returned with complaints of a diffuse erythematous burning, pruritic, maculopapular, non-evanescent rash caused by a severe reaction to sulfasalazine, with a skin biopsy revealing urticarial vasculitis, with probable DRESS (drug reaction with eosinophilia and systemic symptoms) syndrome. This was treated with topical and short-course oral steroids, intravenous antibiotics, and Janus kinase (JAK) inhibitor, improving her condition remarkably. To the best of our knowledge, there are no prior reports of a case of AOSD with normal ferritin levels that also exhibited a severe reaction to sulfasalazine, compounded by complications due to NSAIDs (nonsteroidal anti-inflammatory drugs) and steroid use. This rarity distinguishes our case report.

## Introduction

Adult-onset Still's disease (AOSD) was initially described in children by George F. Still in 1897. However, the disease was predominantly overlooked until Bywaters described it in 1971. AOSD is a rare systemic auto-inflammatory disorder with an unknown cause. It is distinguished by a clinical triad of persistent fever, polyarthritis, and rash [1].

The condition has a bimodal age distribution of 15-25 and 36-46 years of age but mainly affects young individuals [2]. The uncertainty in the presentation and the lack of serologic markers make AOSD diagnosis challenging and is, hence, a diagnosis of exclusion. Given the limitations, the Yamaguchi and Fautrel criteria remain the most commonly used diagnostic tools in clinical practice [3].

Nonsteroidal anti-inflammatory drugs (NSAIDs), corticosteroids (CS), and conventional synthetic disease-modifying antirheumatic drugs (csDMARDs) are mainly used for treatment. In certain instances, biological therapy, such as interleukin inhibitors, tumor necrosis factor-alpha, or Janus kinase (JAK) inhibitors, is administered in refractive cases [4].

Herein, we aim to describe a case of AOSD presenting with normal ferritin levels and a severe drug reaction to sulfasalazine with complications from the use of NSAIDs and steroids, which are the primary treatment modalities for this condition.

## Case presentation

**Figure 1:**
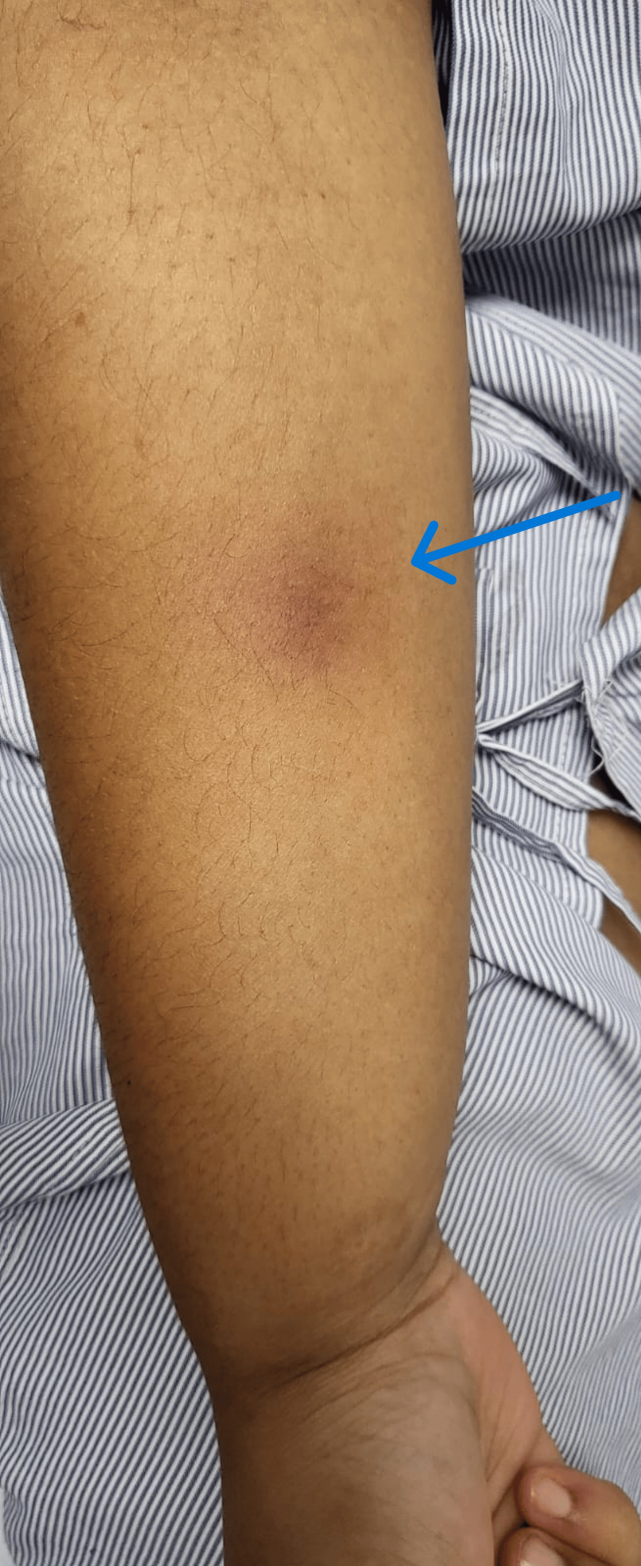
An evanescent, non-pruritic macular rash over the patient's right distal hand, visible during febrile episodes only (arrow).

A 26-year-old female patient presented with a history of polyarthralgia for one month involving the small and the large joints symmetrically, associated with multiple episodes of non-bilious and non-blood-stained vomiting, sore throat, and fever, present for over three weeks, with no prior history or family history of similar episodes. The fever episodes are low-grade, intermittent, not associated with chills or rigors, and relieved after taking antipyretics. However, it is associated with an evanescent, non-pruritic macular rash, mainly over the patient's distal hands (Figure 1).

The patient was treated for possible viral arthritis at another outside hospital with analgesics including aceclofenac 100 mg + paracetamol 325 mg, twice daily, for two weeks, and etoricoxib 90 mg, once a day, for one week, with no significant improvement. Prednisolone, 40 mg once daily, was then added. She gradually developed bilateral distal extremity edema and vomiting over the next few days, which prompted her for a consultation.

The examination indicated an obese female with bilateral distal extremity pitting edema, blood pressure measuring 140/90 mmHg, and no fever at presentation, as she had taken an antipyretic pill before her consultation. Her throat was mildly congested, and occipital and a few cervical lymph nodes were palpable; mild hepatomegaly was appreciated. The musculoskeletal examination was significant for tenderness at the joints of bilateral wrists, bilateral interphalangeal joints of the hands and feet, bilateral knees, and bilateral shoulders. The skin examination showed a few non-pruritic macular rashes, mainly over the patient's distal hands. However, other systemic examinations were unremarkable. During her hospital stay, the patient had multiple episodes of fever in a day, with a recorded highest temperature of 104°F.

Hematological investigations were significant for leukocytosis, mild anemia, elevated C-reactive protein (CRP), high erythrocyte sedimentation rate (ESR), and elevated serum creatinine. A peripheral smear suggested microcytic hypochromic anemia with leukocytosis. Her urine routine detected ketones, likely starvation ketosis, as her glycated hemoglobin was normal. Normal ferritin, coagulation profile, serum cortisol, serum electrolytes, and liver enzymes were noted, as seen in Table 1. The chest X-ray was normal. An ultrasound of the abdomen and pelvis suggested mild hepatomegaly with minimal ascites.

**Table 1:** Comprehensive blood test report

Parameter	Values	Range
White Blood Cell Count	12.80 thou/µL	4.0-10.0 thou/µL
Red Blood Cell Count	4.08 mil/µL	3.8-4.8 mil/µL
Hemoglobin	10.8 g/dL	12.0-15.0 g/dL
Neutrophils	85%	40-80%
Lymphocytes	23%	20-40%
Eosinophils	02%	1-6 %
Platelet Count	288 thou/µL	150-410 thou/µL
Serum Creatinine	1.32 mg/dL	0.60-1.10 mg/dL
Serum Urea	39 mg/dL	21-43 mg/dL
Prothrombin Time	13.0 seconds	12.70-14.60 seconds
INR (International Normalized Ratio)	1.02 ratio	0.00-1.40 ratio
CRP (C-reactive Protein)	26.3 mg/L	3-10 mg/L
TSH (Thyroid-Stimulating Hormone)	1.20 µIU/mL	0.270-4.200 µIU/mL
Hba1c (Glycated Hemoglobin)	5.8%	<6.2%: Normal; 6.3-7.0%: Good control; 7.1-8.0%: Fair control; >8.0%: Poor control
Serum Iron	13 µmol/L	14-32 µmol/L
Serum Transferrin	295 mg/dL	204-360 mg/dL
Serum Ferritin	14.10 mcg/L	11-307 mcg/L
Total Iron Binding Capacity	365 mcg/dL	240-450 mcg/dL
Serum Cortisol 8 a.m.	18 mcg/dL	5-23 mcg/dL
Serum Cortisol 4 p.m.	7 mcg/dL	3-13 mcg/dL
Erythrocyte Sedimentation Rate (ESR)	18 mm/hour	0-15 mm/hour
Direct Bilirubin	0.27 mg/dL	0.00-0.40 mg/dL
Total Bilirubin	0.46 mg/dL	0.3-1.2 mg/dL
Aspartate Amino Transferase (AST/SGOT)	18 U/L	0.0-31.0 U/L
Alanine Aminotransferase (ALT/SGPT)	10 U/L	0.0-34.0 U/L
Alkaline Phosphatase	47 U/L	42-98 U/L
Gamma-Glutamyl Transferase	7 U/L	0.0-38.0 U/L
Serum Sodium	136 mmol/L	135-145 mmol/L
Serum Potassium	4 mmol/L	3.6-5.0 mmol/L
Serum Chloride	105 mmol/L	98-107 mmol/L

The infectious workup was unremarkable, including a throat swab, urine culture, blood culture, and tests (Table 2).

**Table 2:** Infectious workup profile HIV: human immunodeficiency virus

Parameter Name	Result	Method
HIV-1 Antibodies	Non-reactive	Rapid Card Test
HIV-2 Antibodies	Non-reactive	Rapid Card Test
Hepatitis B Surface Antigen	Non-reactive	Rapid Card Test
Hepatitis C Antibodies	Non-reactive	Rapid Card Test
Chikungunya	Non-reactive	Rapid Card Test
Dengue	Non-reactive	ELISA
Weil-Felix	Non-reactive	Agglutination Test
Leptospira	Non-reactive	Microagglutination Test
Scrub Typhus	Non-reactive	ELISA
Mycoplasma pneumoniae	Non-reactive	ELISA
Antistreptolysin O	Non-reactive	ELISA

The workup for autoimmune and inflammatory etiology was unremarkable (Table 3).

**Table 3:** Autoimmune profile

Antinuclear Antibody (ANA) Profile Serum	Result	Method
nRNP/Sm (Nuclear Ribonucleoprotein)	Negative	ELISA
Sm (Smith)	Negative	ELISA
SS-A (Sjogren's Syndrome A)	Negative	ELISA
Ro-52 (TRIM21)	Negative	ELISA
SS-B/La (Sjogren's Syndrome B)	Negative	ELISA
Scl-70 ( DNA Topoisomerase I)	Negative	ELISA
PM-Scl (Polymyositis/Scleroderma)	Negative	ELISA
Jo-1 (Histidyl-tRNA Synthetase)	Negative	ELISA
CENP-B (Centromere B)	Negative	ELISA
PCNA (Proliferating Cell Nuclear Antigen)	Negative	ELISA
ds (double-stranded) DNA	Negative	ELISA
Nucleosomes	Negative	ELISA
Histones	Negative	ELISA
Rib-P (Ribosomal P) Protein	Negative	ELISA
AMA-M2 (Antimitochondrial M2)	Negative	ELISA
Rheumatoid Factor	Negative	ELISA
Anti-CCP (Cyclic Citrullinated Peptide)	Negative	ELISA

Given her clinical features and review of the laboratory values, in the absence of other identifiable causes, she was diagnosed with AOSD using the Yamaguchi criteria. As the patient had no past or family history of drug allergies, she was discharged on hydroxychloroquine 200 mg twice daily, sulfasalazine 500 mg twice daily, and suitable anti-hypertensives.

**Figure 2:**
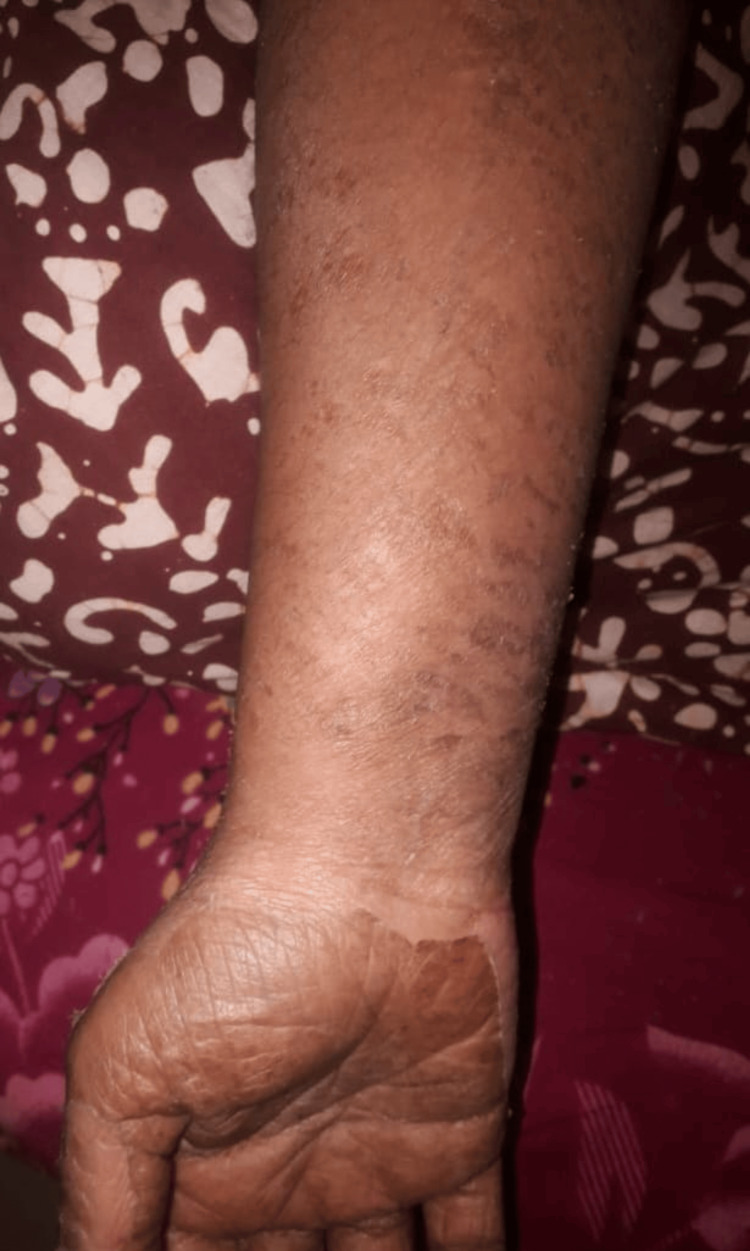
Diffuse erythematous maculopapular, burning, non-evanescent, and itchy rash on the patient's right hand.

**Figure 3:**
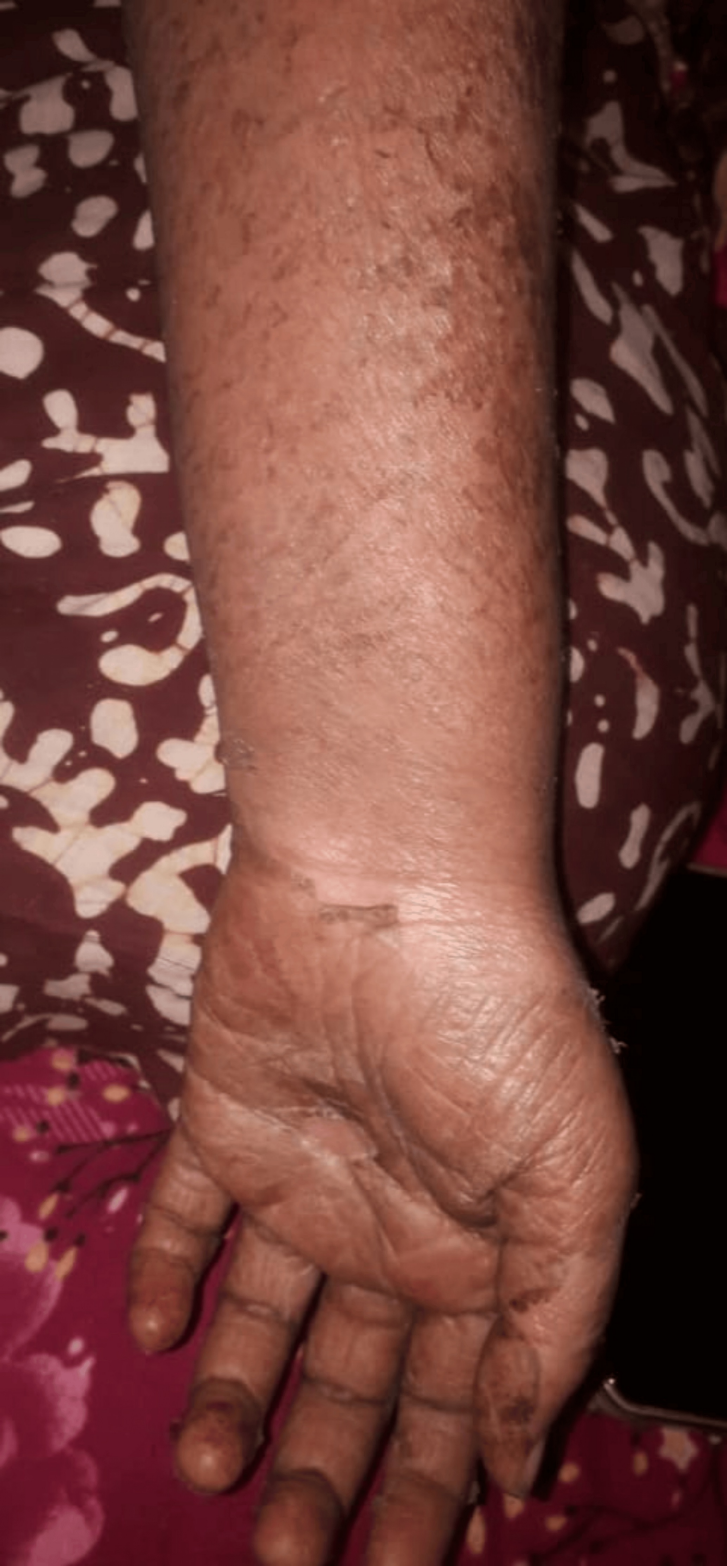
Diffuse erythematous maculopapular, burning, non-evanescent, and itchy rash on the patient's left hand.

Over the next few days, the patient became afebrile, and the arthralgia improved significantly. The patient subsequently presented one week post-discharge with diffuse erythematous maculopapular, burning, non-evanescent, and itchy rash involving her entire body (Figures 2, 3). The rash was associated with fever (103°F), peripheral smear suggesting microcytic hypochromic anemia with leukocytosis and a few atypical lymphocytes, facial edema, distal extremities edema, and cervical and occipital lymphadenopathy.

**Figure 4:**
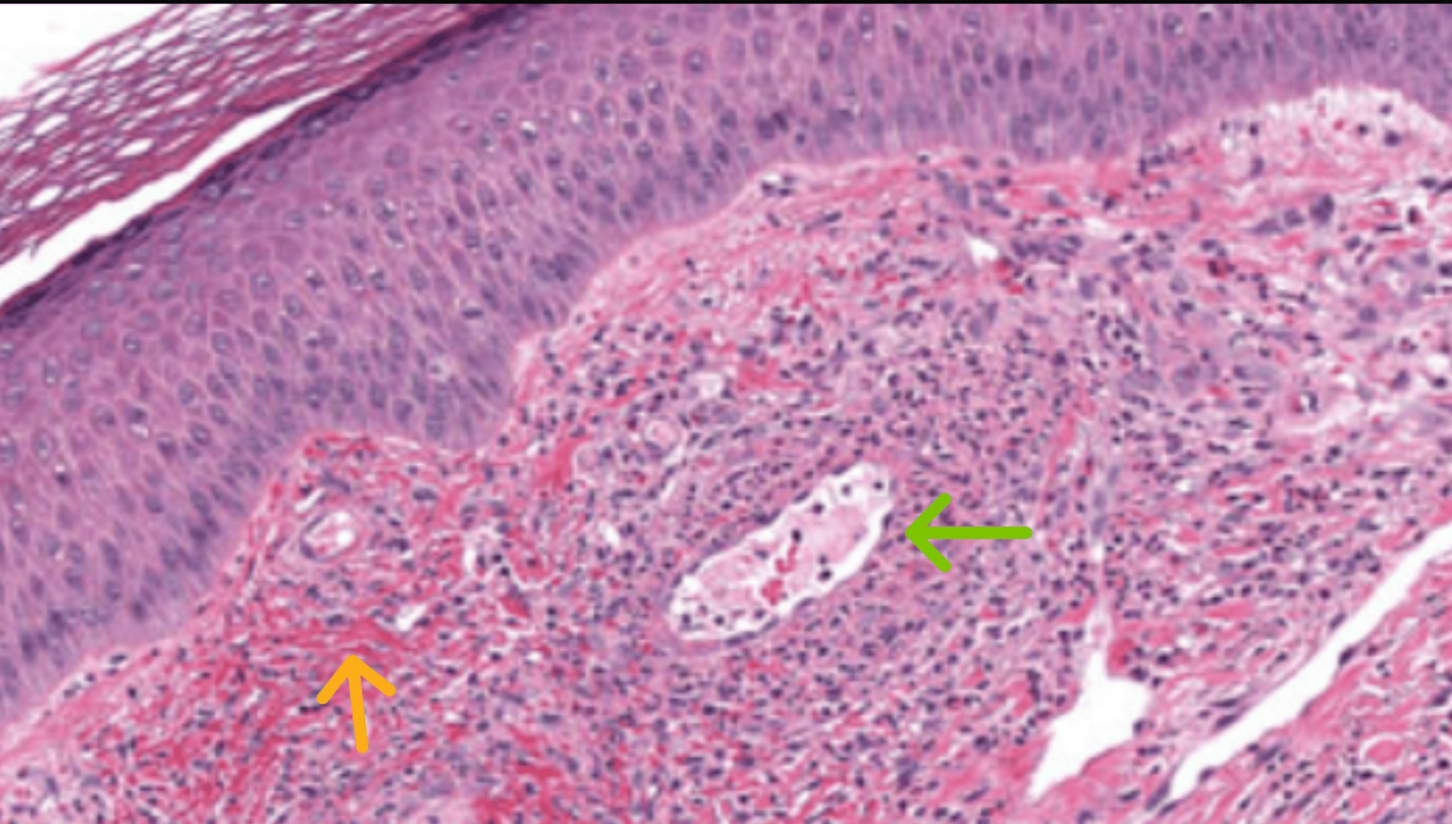
Microscopic examination of the skin biopsy showing fused rete ridges and mild spongiosis, with the superficial dermis having perivascular lymphocytic infiltrates (green arrow), along with rare neutrophils. The surrounding stroma has extravasated red blood cells (orange arrow) and a few karyorrhectic debris. The deeper dermis is within normal limits and shows normal-appearing adnexal structures. No neutrophilic collections. No epidermal necrosis or atypia. Consistent with urticarial vasculitis.

It was suspected to be a drug reaction to sulfasalazine, and immediately, the drug was stopped. Owing to the additional symptoms associated with the rash, a diagnosis of probable DRESS (drug reaction with eosinophilia and systemic symptoms) syndrome was made. The rash was treated with antihistamines to provide the patient with symptomatic relief from itching. A dermatology opinion was sought, and a skin biopsy was ordered, which revealed urticarial vasculitis (UV) (Figure 4).

**Figure 5:**
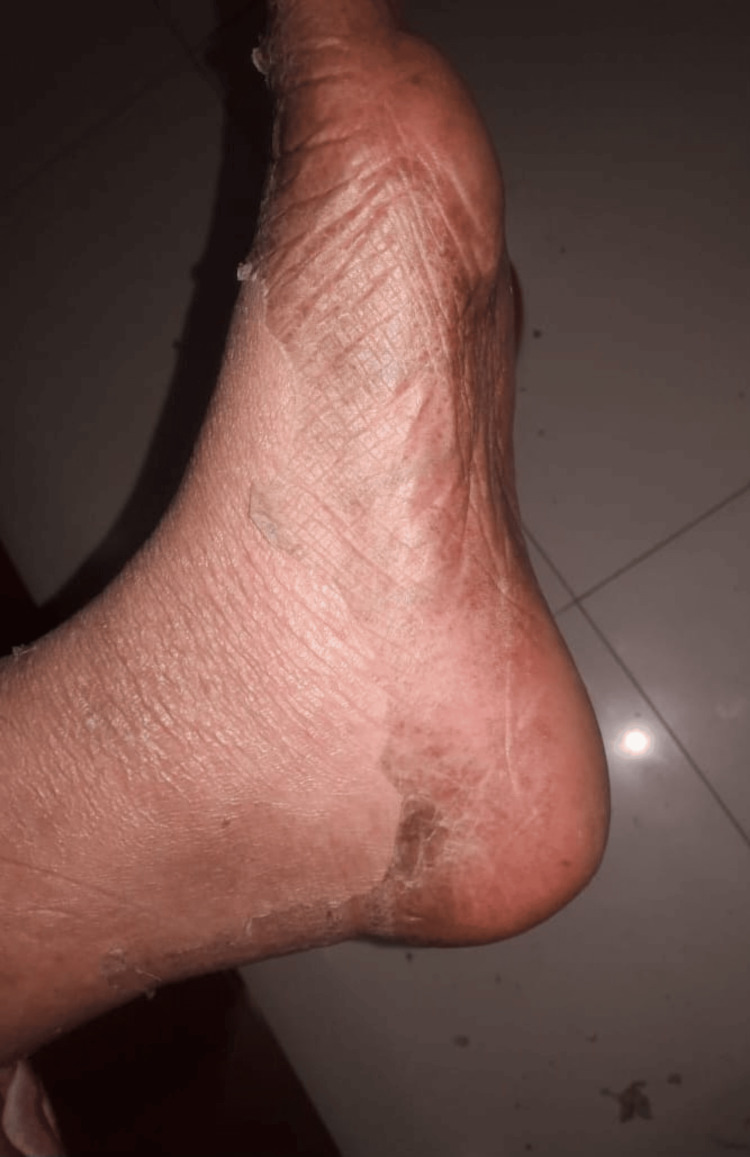
Rashes resolving with peeling and collarette of scales. Plantar and medial aspects of the foot.

**Figure 6:**
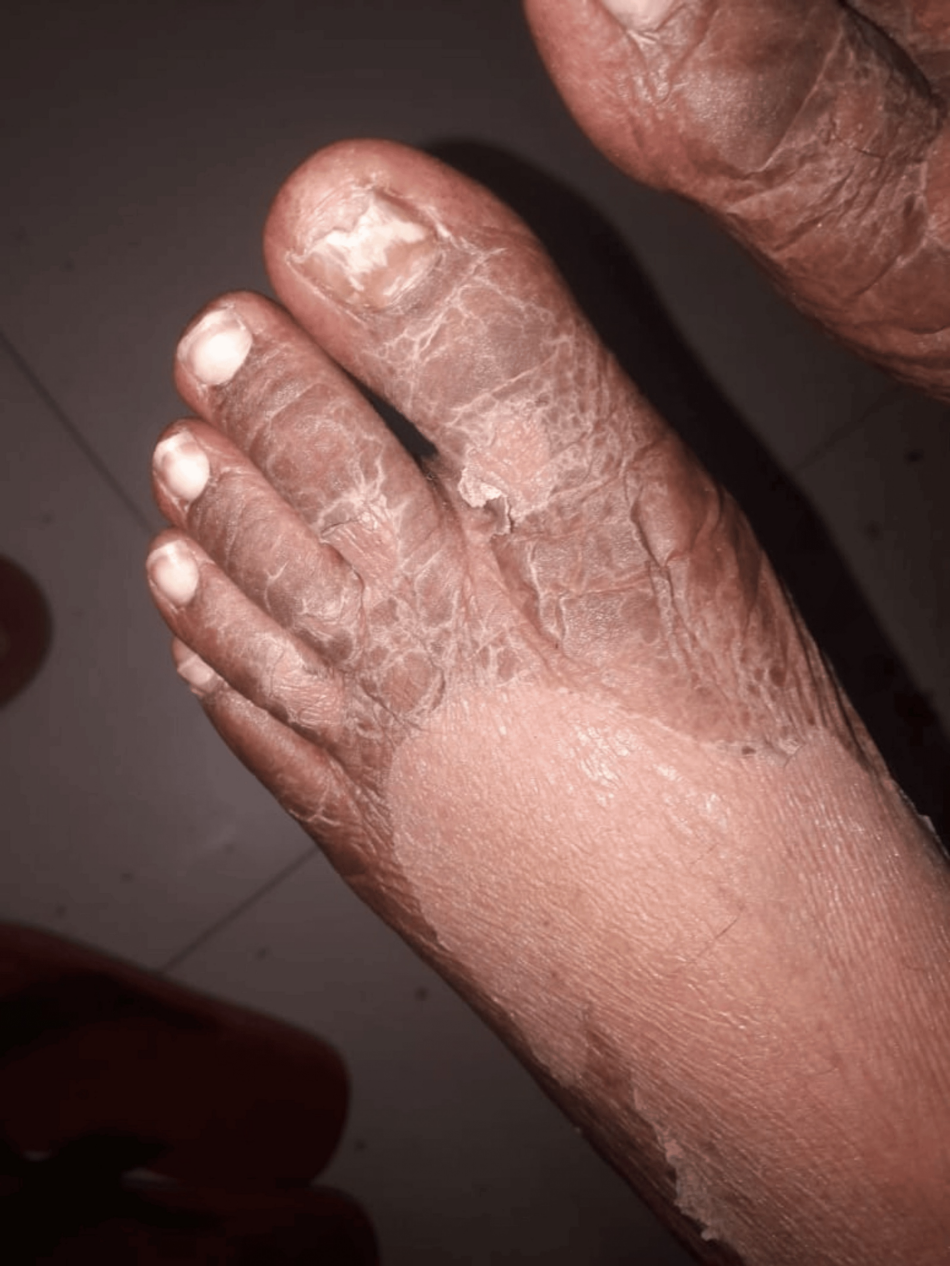
Rashes resolving with peeling and collarette of scales. Dorsal aspect of the foot.

The patient was then treated with topical steroids, low-dose oral steroids, a short course of intravenous antibiotics (to prevent secondary bacterial skin infection), and a JAK inhibitor, tofacitinib, which provided significant improvement. The rashes started resolving with peeling and collarette of scales (Figures 5, 6). The steroids were successfully tapered off over the next few weeks, and her symptoms were completely resolved. At present, she is on hydroxychloroquine 200 mg.

## Discussion

Still's disease is a systemic inflammatory response disorder with an unclear etiopathogenesis. Its symptoms include fever, rash, sore throat, and arthralgia. However, patients may also present with other clinical features, such as sore throat, lymphadenopathy, serositis, hepatomegaly, and splenomegaly [1].

Although AOSD is a diagnosis of exclusion, clinicians can use the Yamaguchi [5] or Fautrel [6] criteria to aid in the diagnosis. The former criteria require that five or more criteria be met, with at least two major criteria, and infection, malignancies, and other rheumatic diseases ruled out. It has a sensitivity of 96.2% and a specificity of 92.1% [5]. The latter criteria require at least four or more major or three major and two minor criteria to be met. It has a sensitivity of 80.6% and a specificity of 98.5% [6].

Our patient's clinical characteristics met the Yamaguchi criteria, with ferritin levels within the normal range, as detailed in Table 4. Conventionally, elevated serum ferritin levels are considered a powerful diagnostic marker for AOSD [7]. However, multiple well-documented cases of AOSD have been reported in which ferritin levels remain unaltered, suggesting the existence of alternative underlying mechanisms [8], thereby rendering it non-pathognomonic for AOSD diagnosis [9].

**Table 4:** Yamaguchi criteria for adult-onset Still's disease (AOSD). Our case is positive for the diagnosis of AOSD For a positive diagnosis, five or more criteria must be met, with at least two major criteria, and infection, malignancies, and other rheumatic diseases must be ruled out [5].

Yamaguchi Criteria	Result
Major criteria	
Fever ≥39°C lasting ≥1 week	Present
Arthralgia or arthritis lasting ≥2 weeks	Present
A nonpruritic macular or maculopapular skin rash that is salmon-colored and usually found over the trunk or extremities during febrile episodes	Present
Leukocytosis (≥10,000/mL), with ≥80 % granulocytes	Present
Minor criteria	
Sore throat	Present
Lymphadenopathy	Present
Hepatomegaly or splenomegaly	Present
Abnormal liver function studies, particularly elevations in aspartate and alanine aminotransferase and lactate dehydrogenase levels	Not present
Negative tests for antinuclear antibody and rheumatoid factor	Present
Exclusion criteria: infections, malignancies, and other rheumatic diseases	Excluded

Ferritin is one of the acute phase reactants, with levels often elevated during inflammation [10]. Additionally, it is well established that steroid therapy attenuates the acute-phase reactant response [11]. In the case at hand, the patient had initially received steroid therapy for her polyarthralgia, which may explain the normal ferritin levels observed during admission. Conversely, it could also represent AOSD with normal ferritin levels.

The patient's polyarthralgia was initially treated with corticosteroids and NSAIDs, both of which are known to cause increased blood pressure and fluid retention, as seen in this case [12,13]. NSAIDs cause this by inhibiting the cyclo-oxygenase enzyme, thereby resulting in acute kidney injury [13], as evidenced by elevated serum creatinine levels in our case. The levels subsequently normalized once the NSAIDs were stopped and treatment was initiated.

Our case also presented with diffuse, erythematous, maculopapular, burning, and pruritic rash involving her entire body one week after discharge with sulfasalazine. Recent trends indicate a rise in atypical cutaneous manifestations, observed in approximately 14% of AOSD cases, with various skin manifestations [14]. In our case, the cutaneous presentation is due to a drug reaction to sulfasalazine with biopsy-proven UV. Sulfasalazine is known to have cutaneous side effects ranging from benign, self-limiting rashes to more severe manifestations such as leukocytoclastic vasculitis [15], which is a feature seen in UV [16]. UV can also be one of the extrapulmonary manifestations of *Mycoplasma pneumoniae*, which can be associated with other symptoms mimicking AOSD [17]. However, our case was negative for its serology. Drug-induced vasculitis was hence strongly suspected as the cause only after other causes of UV were ruled out.

Such side effects of sulfasalazine are more frequently observed in patients with AOSD, ranging from mild symptoms such as nausea, vomiting, abdominal pain, facial flushing, and rashes to more severe reactions, including hypotension, myelosuppression, and even death [18]. Sulfasalazine remains a viable treatment option unless contraindications or significant adverse events arise. Given that some adverse effects have been reported in AOSD patients [18], this should not entirely preclude its use in the treatment, provided careful monitoring is in place.

The cutaneous presentation after sulfasalazine use, along with other features such as fever, enlarged lymph nodes, and blood count abnormality in our case, shines light on DRESS syndrome [19]. Hence, the European Registry for Severe Cutaneous Adverse Reactions (RegiSCAR) criteria were applied, a scoring system for classifying DRESS cases as definite, probable, possible, or no case [20]. Our case belongs to a probable case of DRESS syndrome (Table 5).

**Table 5:** The European RegiSCAR scoring system [20] for diagnosing DRESS syndrome A diagnosis of DRESS syndrome is categorized as follows: definite (score > 5), probable (score 4–5), possible (score 2–3), or ruled out (score < 2), according to the score obtained. Our case records a score of 4, categorizing it as a probable case of DRESS syndrome. * Maculopapular rash and two of the following: (1) facial edema; (2) psoriasiform desquamation; (3) infiltrated skin lesions; (4) purpuric lesions involving areas other than legs. Our case had facial edema and psoriasiform desquamation with maculopapular rash.

Criteria	Score				Result of Our Case
	-1	0	+1	+2	
Fever ≥ 38.5°C (core) or ≥ 38°C (axillary)	No/Unknown	Yes	-	-	Yes (Score: 0)
Enlarged lymph nodes (≥2 sites, >1 cm)	-	No/Unknown	Yes		Yes (Score:+1)
Eosinophils	-	No/Unknown	700-1490	≥1,500	No (Score: 0)
Eosinophils, if leukocytes <4 × 10^9^/L	-	No/Unknown	10.0–19.9%	≥20%	No (Score: 0)
Atypical lymphocytes	-	No/Unknown	Yes	-	Yes (Score:+1)
Skin rash extent (>50% body surface area)	-	No/Unknown	Yes	-	Yes (Score:+1)
Skin rash suggesting DRESS*	No	Unknown	Yes	-	Yes (Score:+1)
Biopsy suggesting DRESS	No	Yes/Unknown	-	-	No (Score: -1)
One organ involvement	-	No/Unknown	Yes	-	No (Score: 0)
Two or more organ involvement	-	No/Unknown	-	Yes	No (Score: 0)
Resolution in ≥15 days	No/Unknown	Yes	-	-	Yes (Score: 0)
At least 3 biological negative investigations done to exclude other causes	-	No/Unknown	Yes	-	Yes (Score:+1)

Our case emphasizes the fact that AOSD should always be considered a possibility in a patient presenting with arthralgia, pyrexia of unknown origin, and a rash, irrespective of the ferritin value. A diagnosis of AOSD can be made even with normal ferritin levels. To the best of our knowledge, a case of AOSD with normal ferritin levels, developing a severe reaction to the use of sulfasalazine, and complications secondary to the use of NSAIDs and steroids have never been reported, which distinguishes this case.

## Conclusions

This case highlights the variability and complexity of the clinical presentation of AOSD. It underscores the need for clinicians to consider AOSD as a differential diagnosis in patients presenting with fever, arthralgia, and rash, even when ferritin levels are not elevated. Furthermore, the case emphasizes the potential adverse reactions to treatment, particularly sulfasalazine, which led to biopsy-proven UV and a probable DRESS syndrome diagnosis. The clinical course was further complicated by the side effects of NSAIDs and steroid therapy. This case, with its unique constellation of symptoms and reactions, serves as a valuable contribution to the existing body of knowledge on AOSD, emphasizing the need for personalized treatment approaches, considering the patient's idiosyncrasies, and careful monitoring of patients when on therapeutic agents.
